# Human stem cells harboring a suicide gene improve the safety and standardisation of neural transplants in Parkinsonian rats

**DOI:** 10.1038/s41467-021-23125-9

**Published:** 2021-05-27

**Authors:** Isabelle R. de Luzy, Kevin C. L. Law, Niamh Moriarty, Cameron P. J. Hunt, Jennifer C. Durnall, Lachlan H. Thompson, Andras Nagy, Clare L. Parish

**Affiliations:** 1 The Florey Institute of Neuroscience and Mental Health, The University of Melbourne, Parkville, VIC Australia; 2 Australian Regenerative Medicine Institute, Monash University, Melbourne, VIC Australia; 3 Lunenfeld-Tanenbaum Research Institute, Mount Sinai Hospital, Toronto, ON Canada

**Keywords:** Regeneration and repair in the nervous system, Regeneration

## Abstract

Despite advancements in human pluripotent stem cells (hPSCs) differentiation protocols to generate appropriate neuronal progenitors suitable for transplantation in Parkinson’s disease, resultant grafts contain low proportions of dopamine neurons. Added to this is the tumorigenic risk associated with the potential presence of incompletely patterned, proliferative cells within grafts. Here, we utilised a hPSC line carrying a FailSafe^TM^ suicide gene (thymidine kinase linked to cyclinD1) to selectively ablate proliferative cells in order to improve safety and purity of neural transplantation in a Parkinsonian model. The engineered FailSafe^TM^ hPSCs demonstrated robust ventral midbrain specification in vitro, capable of forming neural grafts upon transplantation. Activation of the suicide gene within weeks after transplantation, by ganciclovir administration, resulted in significantly smaller grafts without affecting the total yield of dopamine neurons, their capacity to innervate the host brain or reverse motor deficits at six months in a rat Parkinsonian model. Within ganciclovir-treated grafts, other neuronal, glial and non-neural populations (including proliferative cells), were significantly reduced—cell types that may pose adverse or unknown influences on graft and host function. These findings demonstrate the capacity of a suicide gene-based system to improve both the standardisation and safety of hPSC-derived grafts in a rat model of Parkinsonism.

## Abstract (web summary)

Stem cell grafts present a risk of tissue overgrowth/tumors. Here, the authors utilise a human pluripotent stem cell line carrying a FailSafe suicide gene to not only ablate proliferative cells, but through timely gene activation, improve the purity of neural grafts in Parkinsonian rats.

## Introduction

Clinical trials have demonstrated the capacity of transplanted dopamine (DA) progenitors, isolated from foetal ventral midbrain (VM) tissue, to structurally and functionally integrate into the brains of Parkinson’s disease (PD) patients, alleviating motor symptoms for decades^1^. More recently, the generation of VM progenitors from human pluripotent stem cells (hPSCs) has provided an ethical and sustainable alternative donor source. However, while current protocols generate correctly specified VM progenitors with >85% efficiency in vitro^1–4^, the transplantation of these progenitors generate grafts with surprisingly low yields of DA neurons^2,3,5–9^. Studies have now demonstrated that the seemingly low proportion of incorrectly specified cells in culture undergo extensive proliferation following transplantation^1,7,8^, with little knowledge of the impact the vast majority of these non-DA cells will have on graft and host function, or the potential risk for tissue overgrowth/tumours.

In an effort to address these concerns significant attention has focused on the elimination of unwanted and highly proliferative cell types using cell sorting methods prior to transplantation, yet these studies have unfortunately demonstrated varying, and often suboptimal success^7,8,10–12^. Furthermore, this approach fails to safeguard against adverse events such as proliferative cells evading the sorting process or the activation of quiescent stem cells within the graft. Post-transplantation fail-safe approaches, such as suicide gene therapies, have emerged as an alternative option. Several methods now exist, including inducible caspase-9, cytosine deaminase/5-fluorocytine and herpes simplex virus thymidine kinase (HSV-TK)^13^—with the latter system most widely studied and utilised to date. The HSV-TK system, activated by the prodrug ganciclovir (GCV), and targeted at eliminating proliferating cells, has been shown to successfully prevent teratoma growth utilizing either pluripotent stem cell-specific promoters to drive the suicide gene^14–16^, or alternatively cell cycle-dependent promoters to target all proliferating cells^17,18^. Efforts to eliminate proliferating progenitors from within lineage specified cultures and/or transplants have also been explored, with the greatest success observed clinically in the selective elimination of modified donor T lymphocytes in allogeneic hematopoietic stem cell transplants following the development of graft-versus-host disease^19,20^.

While ablation of hPSC-derived neural progenitor cells (NPC) has been achievable in vitro, efforts to eliminate VM NPCs and impact graft size have largely failed when employing a similar GCV regime to that employed for elimination of PSC-derived teratomas^17^. A plausible explanation, and recognised shortcoming of suicide gene therapy, is the risk of silencing or downregulation of the transgene and/or homologous recombination events that have been identified in both PSC cultures and teratomas^17,18,21^. To circumvent this risk, we recently engineered a hPSC line incorporating a transcriptional link between the suicide gene and a gene essential for cell cycle progression (cyclinD1), subsequently referred to as the FailSafe^TM^ hPSC line^18^. We utilized this line to examine the capacity of this system to improve the safety of neural transplants targeted for PD. We demonstrate that GCV administration resulted in significantly smaller grafts at 6 months, yet maintained their full complement of DA neurons, DA innervation, and capacity to restore motor function in Parkinsonian rats, whilst importantly reducing unwanted off-target neural and non-neural populations, including proliferative cells. With hPSC therapies rapidly progressing towards the clinic, these findings have important implications for ensuring the safety and predictability of hPSC-derived DA grafts, targeted at restoring DA transmission in PD patients.

## Results

### VM specification of FailSafe^TM^ hPSCs and validation of suicide gene efficacy

Prior to transplantation, the capacity of the FailSafe^TM^ hPSC line to generate VM progenitors suitable for transplantation was assessed, as well as the efficacy of the suicide system to ablate proliferating neural progenitors in vitro. FailSafe^TM^ hPSCs competently differentiated to VM progenitors, as confirmed by the high proportion of cells co-expressing OTX2+ and FOXA2+ (81.5 ± 5.0%) at D11 of differentiation (Supplementary Fig. 1A, D), with minimal contamination of off-target BARHL1+, PITX2+ diencephalic progenitors (Supplementary Fig. 1B). Ongoing maturation of the cultures until D25 verified their capacity to generate TH+ DA neurons (Supplementary Fig. 1C, D), comparable in efficiency to previously described studies by others and us^1–4,7^.

**Fig. 1:**
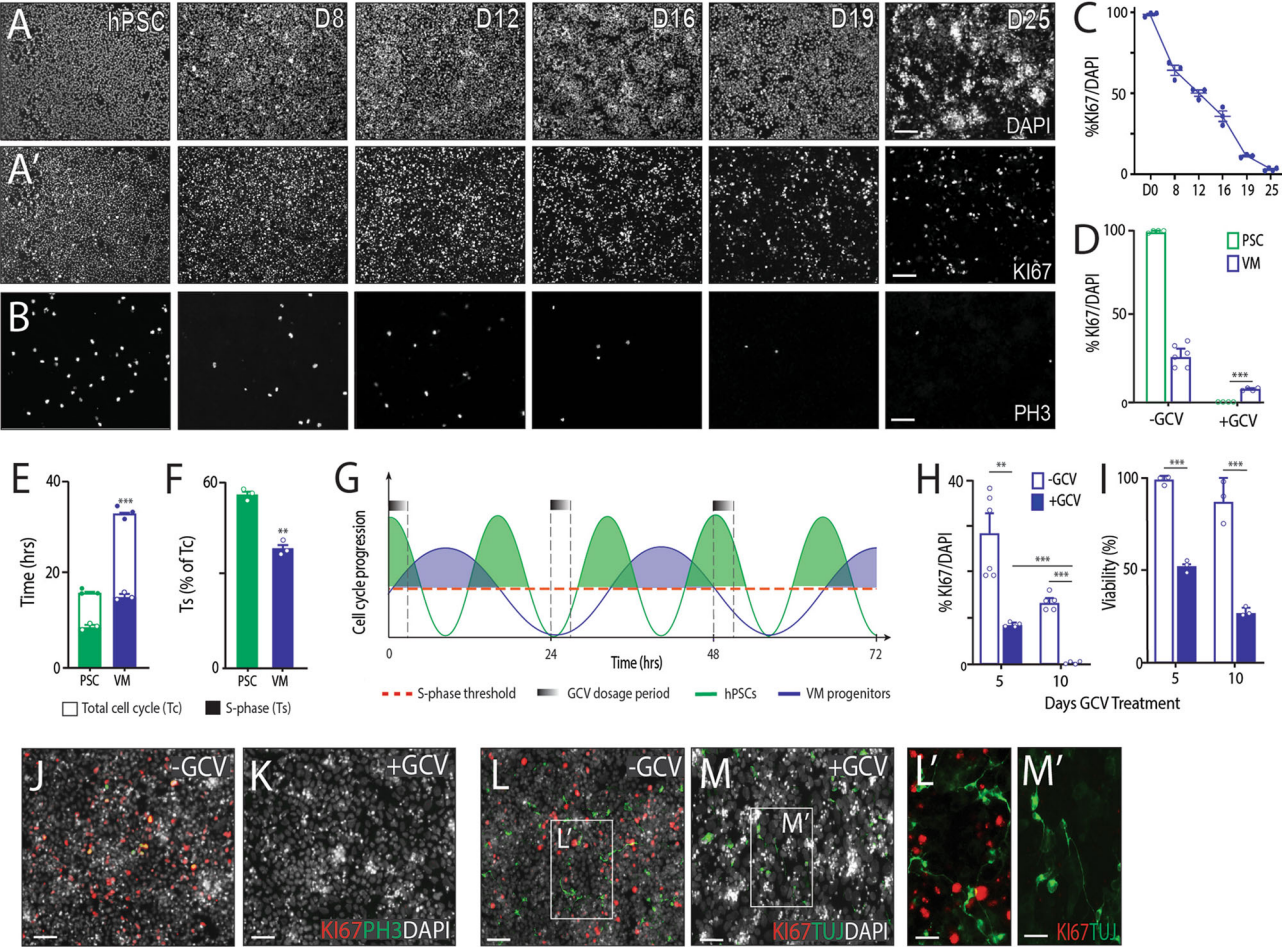
Understanding cell cycle kinetics of PSC and VM progenitors in vitro to optimise ganciclovir-induced cell death. **A**–**B** Representative images showing immunohistochemical labelling against DAPI (**A**), KI67 (**A**’) and PH3 (**B**) tracked cellular proliferation during the differentiation of PSCs into VM dopamine neurons in vitro. **C** The proportion of KI67+ proliferative cells linearly declined from initiation of differentiation (D0) to D25. **D** GCV treatment (10 µM) completely ablated KI67+ cells within undifferentiated PSC (green) but not PSC-derived VM progenitors (blue) cultures. **E** Assessment of cell cycle kinetics revealed prolonged total cell cycle length (Tc, open bars) for VM neural progenitors (blue) compared to undifferentiated PSC (green), yet minimal differences in S-phase (Ts, closed bars). **F** However, as a proportion of total cell cycle length, VM neural progenitors spent significantly less time in S-phase. **G** Graphical representation highlighting the relative differences in cell cycle kinetics of two cell populations—PSCs (green) and PSC-derived VM progenitors (blue). This graphical illustration highlights the increased susceptibility of a highly proliferative cell (e.g. PSC) to GCV (black) elimination, as indicated by the increased amount and proportion of time in S-phase (red dashed line) over a 72-h window. Note an increased number of cell cycles and proportion of time spent in S-phase for PSCs compared to VM progenitors (green vs blue shading). **H** Prolonging GCV treatment from 5 to 10 days (closed blue bars) was sufficient to ablate KI67 + proliferating VM progenitors in vitro. **I** Assessment of viable cells confirming presence of postmitotic cells not affected by GCV treatment. **J**–**K** Representative images showing. immunostaining of cultures for KI67 (red), PH3 (green) and DAPI (white) in the absence (**J**) or presence (**K**) of GCV. **L**–**M** Co-labelling of KI67 (red) and TUJ (green) indicated that some cells remaining after sustained in vitro GCV exposure (19DIV) were post-mitotic neurons (KI67^-^/TUJ^+^). Note the healthy morphology and fibre outgrowth of the TUJ+ neurons even in the presence of GCV (M’). Data are presented as mean values ± SEM. **A**–**C** *n* = 4, **D**, **H** *n* = 6, **E**, **F**, **I** *n* = 3 independent experiments for all cultures conditions. **C** One-way ANOVA with Tukey correction for multiple comparisons. **D**, **E**, **F**, **H**, **I** Multiple student’s *t* test, **F** *t*-test. **p < 0.01, ***p < 0.001. *n* = 3–4 independent cultures per treatment and/or time point. Scale bars: (**A**, **A**’, **B**, **J**–**M**) 100 µm, (**L**’, **M**’) 50 µm. D days in differentiation, GCV ganciclovir, PH3, phosphohistone3, PSCs pluripotent stem cells, VM ventral midbrain.

To determine the capability of the FailSafe^TM^ system to ablate proliferative VM progenitors, we initially tracked the proportion of KI67+ proliferative cells during the VM differentiation. As expected, a progressive decrease in proliferation was observed over time (D0: 98.4 ± 1.2%, D8: 64.2 ± 3.2%, D12: 50.2 ± 2.0%, D16: 35.9 ± 3.3%, D19: 11.3 ± 1.0%, D25: 3.0 ± 0.5%, Fig. 1A–C). To confirm the efficacy of the suicide gene, cultures were treated with GCV at D8—a stage in the differentiation when the cells were sufficiently patterned into VM progenitors. At this time point, cultures showed a high proportion of dividing cells (>60%), yet also comprised a sufficient pool of post-mitotic cells to enable additional assessment of potential drug toxicity. While GCV (10 µM) ablated all PSC in undifferentiated cultures within 5days, only partial ablation (59.2%) of proliferating VM progenitors could be achieved (VM culture: 19.47 ± 3.26% KI67+ cells, VM culture + GCV: 7.93 ± 0.55% KI67, Fig. 1D).

We subsequently questioned whether differences in cell cycle kinetics between PSC and VM neural progenitors may underpin these differences in GCV efficiency, noting that GCV only incorporates into dividing cells during S-phase/DNA replication, and given the existing evidence of reduced S-phase duration with cellular maturity in development^22^. We assessed and compared the cell cycle kinetics of Day 9 VM neural progenitors in comparison to undifferentiated PSCs using a cumulative EdU labelling technique^23^. Total cell cycle length (Tc) significantly increased in maturing VM progenitor cultures (PSCs: 16 ± 0.4 h, VM: 33 ± 0.6 h, Fig. 1E), while the proportion of time spent in S phase (Ts) was significantly reduced (PSCs: 55%, VM: 44%, Fig. 1F, Supplementary Fig. 2). Consequently, when considered over several days, the amount of total time spent in S-phase (above the red line, Fig. 1G) is notably less for VM progenitors compared to PSCs (blue and green shading, respectively, Fig. 1G), and hence reduced susceptibility to GCV ablation. We therefore looked to prolong the duration of GCV administration. Complete elimination of KI67+ and PH3+ VM progenitors could be achieved following 10 days exposure to GCV (Fig. 1H, J, K), with evidence of viable post-mitotic cells (Fig. 1I, K, M), including neurons (Fig. 1M), that were not affected by GCV treatment. Collectively these findings demonstrated the robust differentiation efficacy of the FailSafe^TM^ PSC line into VM progenitors and neurons, and the capacity to successfully ablate proliferative cells in vitro.

### Tracking the proliferation and maturation of VM progenitor grafts to identify the optimal timing for suicide gene activation

While older (D25) differentiated cultures possessed fewer proliferative cells (Fig. 1A–C), and may therefore be considered a safer donor population, numerous rodent and human foetal grafting studies have demonstrated that older VM donor cells are in fact inferior to younger, due to the poor survival of the postmitotic TH+ DA neurons at the time of harvesting and implantation^24–26^. In order to determine the ideal progenitor age for grafting and assessment of the FailSafe™ system, we therefore compared our routinuely utilised D19 human PSC-derived VM donor cells against D25 aged cells, differentiated under our defined culture conditions^4^.

Reflective of in vitro development and maturation, a small proportion of cells (5.6% ± 4.3%) expressed TH+ at D19 of differentiation, increasing to 30.1% ± 3.6% by D25 (Supplementary Fig. 3A–C). Unsurprisingly, upon engraftment, these older D25 VM donor cells, whilst generating grafts of comparable size to D19 progenitors, showed significantly fewer TH+ cells (Supplementary Fig. 3D–G) and reduced capacity to innervate the host striatum (Supplementary Fig. 3E, F, H), thereby justifying the subsequent use of younger D19 donor cells. While the present findings show superior grafting outcomes using younger D19 VM progenitors, other groups have demonstrated functional engraftment using older donor cells^3,11^. Such variable observations highlight the importance of assessing differentiation and grafting outcomes in the context of different hPSC lines and the protocols adopted to fate restrict hPSCs.

**Fig. 2:**
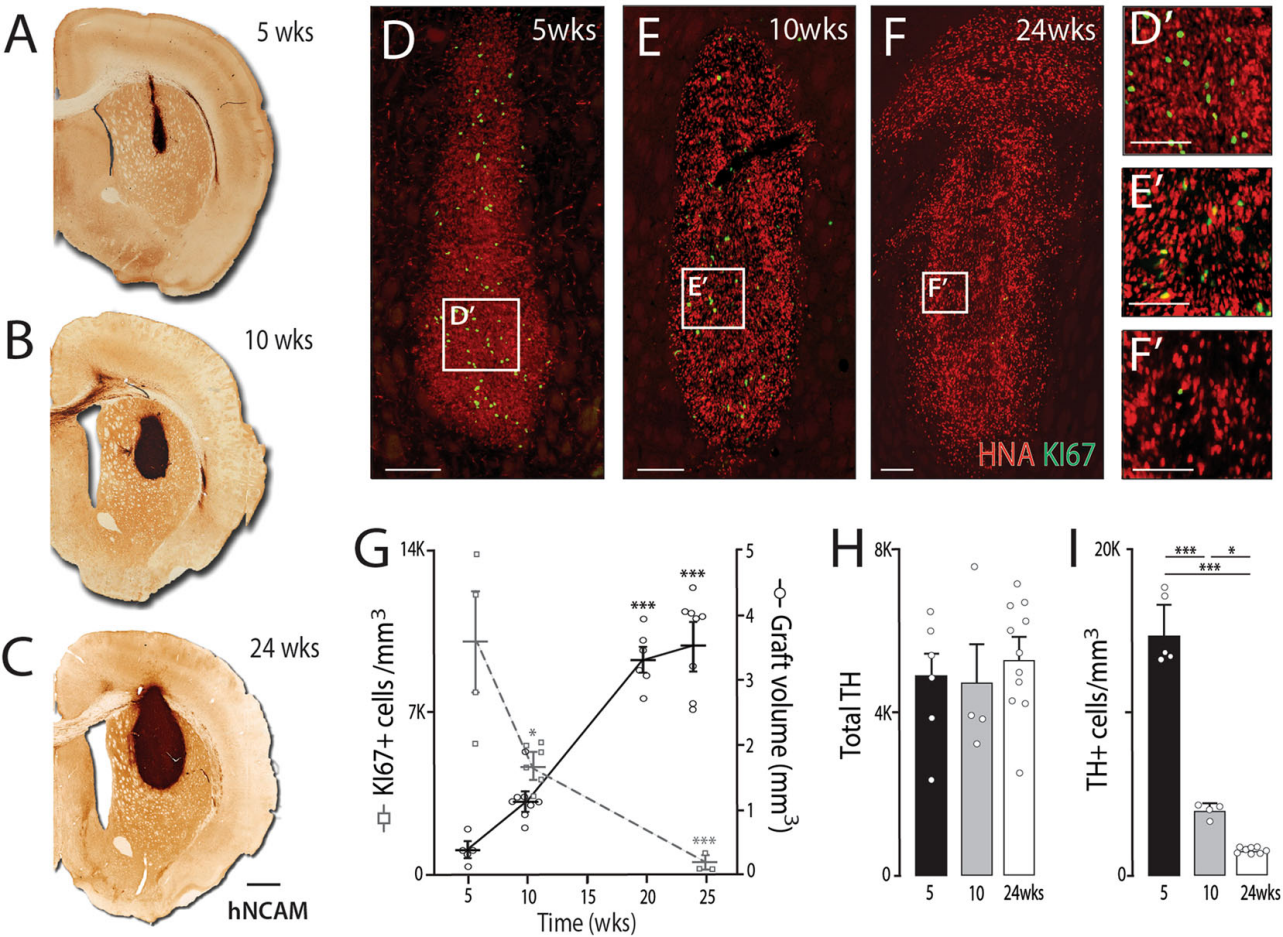
Tracking expansion and DA neurogenesis within VM progenitor grafts. **A**–**C** Coronal sections of the rat brain immunolabeled for human-specific NCAM demonstrating relative graft expansion between (**A**) 5, (**B**) 10 and (**C**) 24 weeks. Images are taken from the middle section of the graft for each representative. **D**–**F** Photomicrographs showing immunohistochemical labelling of grafts with HNA (red) and Ki67 (green). **D**’–**F**’ High magnification inserts from (**D**–**F**) showing the decrease in KI67 proliferative progenitors within grafts over time. **G** Quantitative assessment showing increased graft volume from 5 to 24 weeks (black line), with a progressive decrease in the density of KI67+ proliferating cells (grey line); 5-week old graft (*n* = 6); 10-week graft (*n* = 8); 20-week graft (*n* = 7); 24-week graft (*n* = 8). **H** Quantification of TH+ cells within grafts at varying intervals after implantation highlighted that all DA neurons were born by 5 weeks, **I** yet TH+ cell density significantly reduced over time due to continued graft expansion; 5-week old graft (*n* = 6); 10-week graft (*n* = 4); 24-week graft (*n* = 11). Data are presented as mean values ± SEM. **G**–**I** One-way ANOVA with Tukey correction for multiple comparisons. *p < 0.05, ***p < 0.001. Scale bars: **A**–**C**: 1 mm, **D**–**F** 500 µm, **D**’–**F**’ 200 µm. HNA human nuclear antigen, hNCAM human polysialylated neural cell adhesion molecule, TH tyrosine hydroxylase.

Recent studies have highlighted that proliferation after engraftment arises by not only the DA progenitors, but other cell populations^6–8^. To therefore gain greater insight into the growth kinetics of these D19 VM progenitor grafts, we assessed graft volume and proliferation over a period of 6 months, as well as the timing at which VM progenitors adopted a post-mitotic DA neuron fate. Grafts showed a significant, volumetric expansion over time (5 weeks: 0.4 ± 0.1 mm^3^, 10 weeks: 1.1 ± 0.2 mm^3^, 20 weeks: 3.3 ± 0.2 mm^3^, 24 weeks: 3.5 ± 0.4 mm^3^; Fig. 2A–C, G). KI67 immunolabeling highlighted numerous proliferative cells present at 5 weeks (9,306 ± 1997 KI67+/mm^3^) that progressively decreased in density with time (10 weeks: 4672 ± 629 KI67+/mm^3^; 24 weeks: 1507 ± 230 KI67^+^HNA^+^) and comprising of <0.4% of total cells at 24 weeks (Fig. 2D–G). Interestingly, the total number of DA neurons remained unchanged from 5 to 24 weeks (Fig. 2H, 5 weeks: 4752 ± 708, 10 weeks: 4635 ± 1080, 24 weeks: 5204 ± 467), yet resulted in a significant decrease in DA cell density due to the continued expansion of the graft size (5 weeks: 15,000 ± 1763 TH^+^/mm^3^, 24 weeks: 1,524 ± 83 TH^+^/mm^3^, Fig. 2I). As the full complement of TH+ DA neurons were present by 5 weeks after implantation, this suggests that graft expansion observed beyond this time was a result of the proliferation of poorly specified progenitors, not destined to become DA neurons. Note, whilst we show here that all TH+ DA neurons are present within the FailSafe^TM^ hPSC-derived VM progenitor grafts by 5 weeks, this will require confirmation on a hPSC line-to-line basis and be dependent upon the VM differentiation protocols employed.

### Early and sustained activation of the suicide gene reduced graft size while conserving DA composition and functionality

**Fig. 3:**
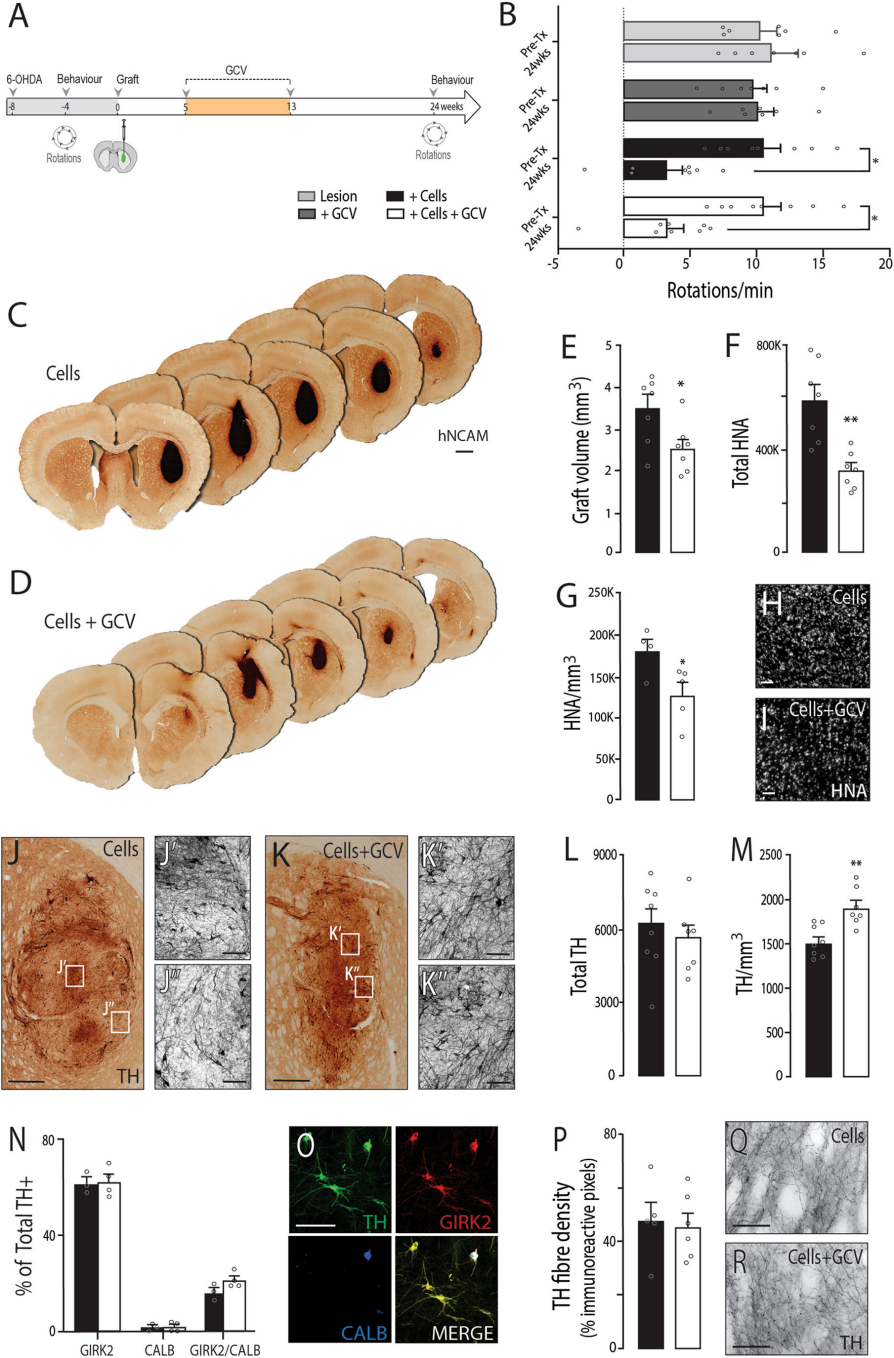
Early and sustained GCV treatment prevented graft expansion without effecting graft function and dopaminergic composition. **A** Schematic overview of in vivo study design. **B** All grafted animals showed restoration of amphetamine-induced rotational asymmetry at 24 weeks, illustrating that GCV treatment had no detrimental effect on graft function. **C**–**D** Photomontage illustrating a coronal representative overview of a VM progenitor graft in the (**C**) absence and (**D**) presence of GCV. **E**–**G** Graphs highlighting that GCV treatment (white bars) generated significantly smaller grafts (**E**), containing fewer cells (**F**) and overall reduced cell density (**G**), compared to untreated grafts (black bars). **H**–**I** Representative images of HNA+ labelled cells within grafts, highlighting the decreased cell density in (**I**) GCV-treated, compared to (**H**) untreated animals. **J**–**K** Photomicrographs showing distribution and density of TH+ cells within grafts in the absence (**J**) and presence (**K**) of GCV treatment, noting increased homogeneity of TH+ cells in grafts exposed to GCV (**J**’, **J**”, **K**’, **K**”). **L** GCV treatment had no effect on TH+ cell number within grafts, **M** but led to a significant increase in TH+ DA neuron density. **N** Quantitative assessment of TH+ neurons that co-express GIRK and/or calbindin showed that GCV treatment did not impact on the capacity of grafted DA neurons to adopt both A9 and A10 mature phenotypes. **O** Representative photomicrograph showing TH+ (green) co-expression together with GIRK2 (red) and calbindin (blue), highlighting the presence of both A9- and A10-like midbrain dopaminergic neurons within the grafts. **P** Quantitative assessment of TH+ fibre density in the dorsolateral striatum. **Q**–**R** High power images illustrating maintained density of TH+ innervation in grafted animals exposed to GCV treatment. Data are presented as mean values ± SEM. (**B**, **E**–**G**, **L**, **M**, **P**: *n* = 7–8 grafts/group; **N**: *n* = 4 grafts/group). **B**, **N** Multiple student’s *t*-tests, **E**–**G**, **L**, **M**, **P** *t*-test. *p < 0.05, **p < 0.01, ***p < 0.001. Scale bars: **C**–**D**: 1 mm, **J**–**K** 500 µm, **J**’, **J**”, **K**’, **K**”, **Q**, **R**: 100 µm. **H**, **I**, **O** 50 µm. GCV ganciclovir, HNA human nuclear antigen, hNCAM human polysialylated neural cell adhesion molecule.

In an effort to improve the safety and purity of the hPSC-derived neural grafts, we aimed to selectively ablate the proliferative cell population responsible for the expansion of the grafts but not destined to become DA neurons. To achieve this, grafted animals were treated with GCV daily from 5 weeks (the earliest time at which the full complement of post-mitotic DA neurons were present within the graft). Given the ongoing growth of these hPSC-derived VM progenitor grafts beyond the neurogenic window of the DA progenitors (notably from 5 to 20 weeks, Fig. 2G), in combination with the knowledge of (i) grafting/teratoma studies of undifferentiated PSCs requiring GCV treatment for 4 weeks to eliminate rapidly dividing cells^18^, (ii) the prolonged cell cycle of VM progenitors, with a proportionately shorter S-phase compared to hPSCs (Fig. 1E, F), and, (iii) the short half-life of the pro-drug GCV (<2 h after intraperitoneal injection in rodents^27^), treatment was maintained for an extended duration of 8 weeks (Fig. 3A).

Rats receiving 6-OHDA lesions were tested for amphetamine-induced motor asymmetry, and only animals showing >5 rotations/min were included in the study. At 24 weeks after grafting both Lesion only (light grey bars) and Lesioned animals treated with GCV (dark grey bars) showed a sustained functional deficit (Fig. 3B). In contrast, grafted animals displayed a significant improvement in motor function, irrespective of exposure to GCV (Fig. 3B, black and white bars), indicating that neither the drug (GCV) nor ablation of proliferative cells had a detrimental effect on the function of DA neurons within the grafts.

Post-mortem assessment revealed surviving grafts in all animals, visualised by the presence of human-specific PSA-NCAM staining (hNCAM), Fig. 3C, D. Grafts in animals treated with GCV, however, were significantly smaller (Cells: 3.5 ± 0.4 mm^3^; Cells + GCV: 2.5 ± 0.2 mm^3^), contained 47% fewer cells, quantified by immunolabeling against human nuclear antigen, HNA (Cells: 577,226 ± 70,530 HNA+ cells, GCV: 306,451 ± 33,365 HNA+ cells), and showed a significant reduction in HNA+ cell density (Fig. 3E–I).

No significant difference in the number of TH+ DA neurons was observed between grafted animals (Cells: 6061 ± 643; Cells + GCV: 5516 ± 612, Fig. 3J–L), supporting the comparable improvement in functional recovery. Consequently, as a result of the significant reduction in graft volume, the density of DA neurons within GCV-treated animals were significantly increased (Cells: 1731 ± 83 TH+ cells/mm^3^, Cells + GCV: 2,206 ± 72 TH+ cells/mm^3^, Fig. 3M) and the distribution of these neurons were notably more homogeneous than grafts not treated with GCV, noting patches of both high and low TH density in non-GCV-treated animals (Fig. 3J’, J”, K’, K”). Importantly, exposure of the graft to GCV had no impact on the maturation of the DA neurons into A9 or A10-like subtypes with >80% of TH+ neurons expressing GIRK2 and/or Calbindin (Fig. 3N, O). Similarly, GCV treatment had no impact on the ability of TH+ DA neurons within the grafts to innervate the host dorsolateral striatum—the primary target of A9-like DA neurons responsible for motor function (Cells: 47.3 ± 6.7%, Cells + GCV: 44.7 ± 5.3% TH+ immunoreactive pixels) (Fig. 3P–R). More detailed assessment revealed comparable TH+ innervation density in GCV-treated and untreated animals, not only across the rostro-caudal axis of the dorsolateral striatum (Supplementary Fig. 4C, D), but similarly across the axis of the ventrolateral striatum (an area responsible for more complex sensorimotor function^28^) and the medial striatum (Supplementary Fig. 4B–D).

In an additional cohort of animals, intended to demonstrate the ability of delayed GCV treatment (from 14 to 22 weeks) to reduce residual proliferative cells within grafts (Supplementary Fig. 5A) we showed no change in graft size yet significantly reduced KI67+ cells (Supplementary Fig. 5B–E). Behavioural assessment at 20 weeks demonstrated that during periods of ongoing GCV treatment (and localised suicide-induced cell death) graft functionality was maintained, with correction of rotational asymmetry comparable to the non-GCV-treated animals (Supplementary Fig. 5F).

### Suicide-induced cell death within neural grafts has no effect on local inflammation

With desired cell death occurring within GCV-treated grafts, as a consequence of suicide gene activation, we assessed the impact on local inflammation. A separate cohort of animals were examined at 10 weeks after transplantation, in the presence or absence of GCV administration from weeks 5 to 10. During this period of tightly regulated GCV-induced apoptosis, treated animals unsurprisingly showed no change in the densities of Iba1+ microglia or GFAP+ reactive astrocytes within the graft core (field of view 1, FOV1) and surrounding host tissue (FOV2 and FOV3), compared to non-GCV-treated grafts (Supplementary Fig. 6A–B, D)—noting that apoptotic cell death minimally evokes an inflammatory response. While Iba1 and GFAP immunoreactivity was elevated within the graft cores (at FOV1) at 10 weeks after grafting compared to the host tissue (at FOV2 and FOV3, Supplementary Fig. 6B–C), Iba1+ microglia levels were significantly reduced by 24 weeks, and more subtly for GFAP astrocytes (Supplementary Fig. 6D–E), indicative of transient inflammatory response to neural grafting that diminishes with time, as described in clinical trials involving human foetal tissue^29^. Importantly, this transient inflammatory response observed at 10 weeks had no impact on the survival or functionality of the TH+ cells within the grafts as the full complement of DA neurons and their ability to reverse motor asymmetry at 24 weeks was observed in GCV-treated animals, comparable to non-GCV treatment (Fig. 3L, B, respectively). While these findings show that cell death within the grafts (as a consequence of suicide gene activation) had a negligible inflammatory effect, one needs to acknowledge the limitations of the model in which these studies were performed. Current preclinical xenografting models (inclusive of athymic animals or immunosuppression) are unable to fully anticipate the immunological response to activation of the gene in human allografts.

### Early suicide gene activation reduced the levels of non-dopaminergic neural and non-neural cells within grafts

Recent transcriptomic and histochemical studies of mature dopaminergic grafts have highlighted their heterogeneous composition including not only other neuronal and neural populations but also non-neural cell types, despite implanting VM progenitors of high purity^30,31^. With smaller grafts, of maintained DA composition, observed following GCV treatment we sought to determine the cell population/s reduced in number or eliminated from the grafts.

**Fig. 4:**
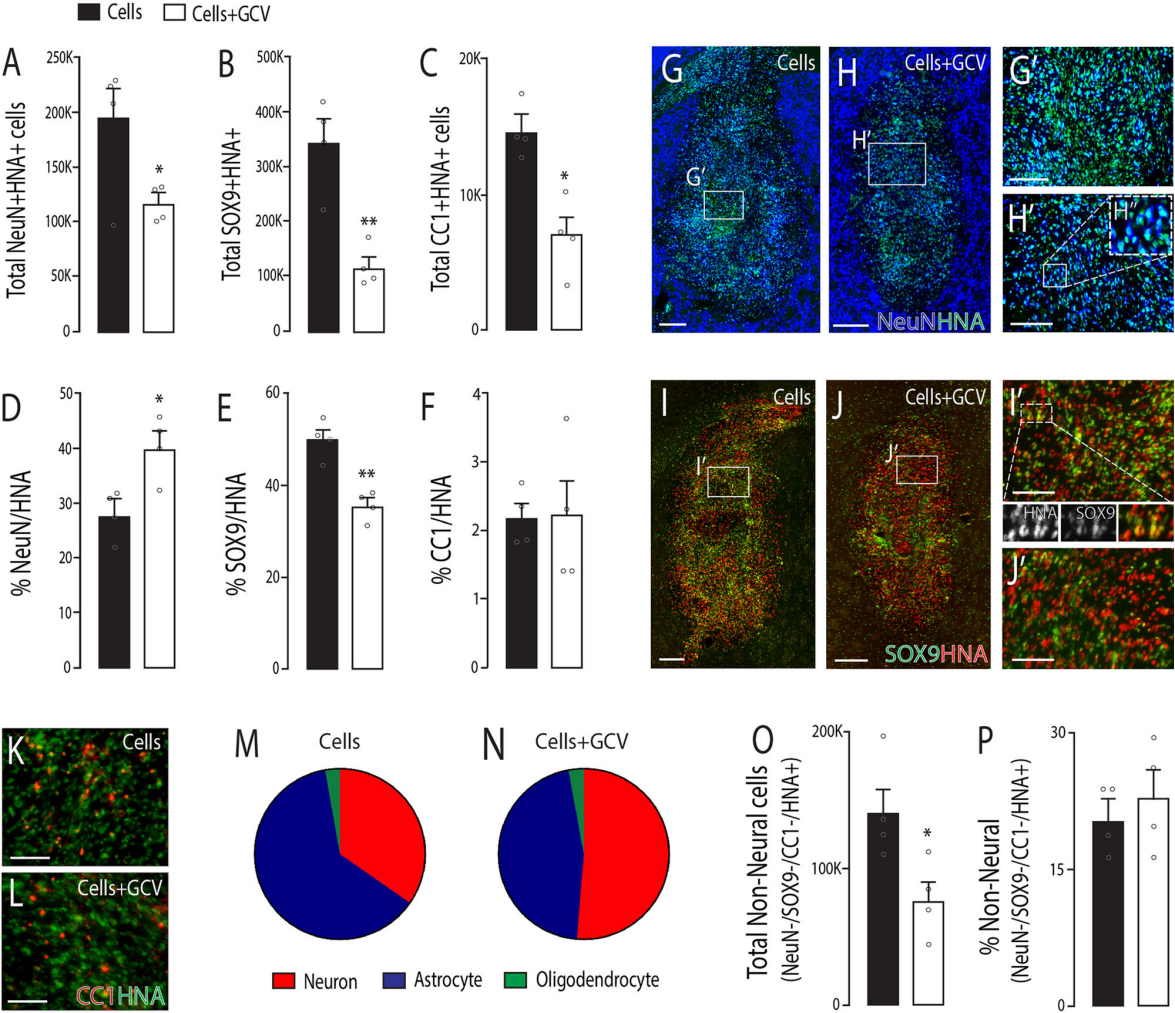
The Failsafe^TM^ suicide system significantly reduced numbers of neural and non-neural cells within VM progenitor grafts. **A**–**C** Quantitative assessment of the total number of NeuN+HNA+ neurons (**A**), SOX9 + HNA+ astrocytes (**B**), and CC1 + HNA+ oligodendrocytes (**C**) within grafts of hPSC-derived VM progenitors in the absence (black bars) and presence (white bars) of GCV treatment. **D**–**F** Proportion of NeuN+ (**D**), SOX9+ (**E**) and CC1+ cells (**F**) cells within the grafts. **G**–**H** Representative photomicrographs illustrating NeuN+ (blue) and HNA+ (green) cells within grafts. **G**’–**H**’ Higher magnification images from (**G**–**H**), highlighting the increase in neuronal specification within the grafts treated with GCV. **I**–**J** Sample images showing SOX9+ (green) and HNA (red) immunolabeling within grafts and (**I**’–**J**’) inserts depicting the visible reduction in the proportion of SOX9+ (green cells) in the presence of GCV. **K**–**L** Representative images of CC1+ (red) and HNA+ (green) cells within GCV-treated and untreated grafts. **M**–**N** Relative proportion of neural subtypes within grafts in the absence (**M**) and presence (**N**) of GCV treatment, noting the reduction in astrocytes and increase in the neuronal specification in GCV-treated grafts. **O** Total number and **P** proportion of non-neural cells within hPSC-derived VM progenitor grafts ± GCV treatment, noting the significant reduction in non-neural cells with GCV-treatment. Data are presented as mean values ± SEM. **A**–**F**, **O**, **P** student’s *t*-tests. *p < 0.05, **p < 0.01; *n* = 4 grafts/group. Scale bars: **G**–**J**: 500 µm, **G**’, **H**’, **I**’, **J**’, **K**, **L**: 100 µm. HNA human nuclear antigen, GCV ganciclovir.

Assessment of the neural composition of the grafts revealed a significant reduction in total number of NeuN + HNA+ neurons, SOX9 + HNA+ astrocytes and CC1 + HNA+ oligodendrocytes (Fig. 4A–C). Interestingly, when assessed as a proportion of the total HNA+ cells within the grafts, a significant increase in the neuronal specification was observed (Cells: 29.7 ± 2.0%; Cells + GCV: 39.4 ± 3.4%, Fig. 4D, G, H). While we observed a significant increase in the proportion of TH+ DA neurons in the graft (Fig. 3M), assessment of other neuronal populations revealed no change, with GABA+ cells contributing to ~5% of neurons in the grafts (Supplementary Fig. 7A–C), ChAT+ cholinergic notably sparse (Supplementary Fig. 7D), and other neuronal populations visibly absent (including DBH+ adrenergic/noradrenergic and 5HT+ serotonergic neurons).

Whilst no change in the proportion of CC1+ oligodendrocytes was observed (indicating a relatively comparable ablation of this cell population, Fig. 4F, K, L), most notable was the significant decrease in the percentage of SOX9+ astrocytes by GCV treatment (Cells: 49.5% ± 2.4; Cells + GCV: 35.2% ± 1.9, and a 33% reduction in total SOX9 + HNA+ cells), Fig. 4B, E, I–J. Figure 4M, N shows the relative neural contribution of neurons, astrocytes and oligodendrocytes within the grafts, highlighting that GCV treatment enriched for neurons, by suppressing gliogenesis.

Assessment of the non-neural component of the graft (HNA+ but not expressing CC1, SOX9 nor NeuN) revealed a significant (46.0%) decrease in the total number of these cells (Fig. 4O), proportionate to the decrease in cells within the graft, and reflected by no change in the proportion of non-neural cells (Fig. 4P). Whilst recent transcriptomic assessment has shown the presence of a high proportion of leptomeningeal-like COL1A1+ cells to be present within VM grafts^31^, these cells were largely absent in the present grafts (yet could be observed within blood vessel-like structures within hPSC-derived teratomas, Supplementary Fig. 8A–B). In contrast, vascularisation of the grafts was predominantly from host-derived vascular cells, as revealed by immunoreactivity against the rat endothelial cell antigen-1 (RECA), with labelling not different between the graft groups (Supplementary Fig. 8A, C–E). Of significant importance, at 6 months after transplantation, proliferating cells of graft origin (KI67 + HNA+, and confirmed by confocal imaging) were notably rare, and significantly reduced (41%) in the presence of GCV treatment (Cells: 1667 ± 367, Cells + GCV: 993 ± 152, Supplementary Fig. 8F), and typically observed in close proximity to infiltrating vasculature (Supplementary Fig. 8F, F’).

## Discussion

The low proportion of DA neurons within hPSC-derived VM progenitor grafts observed across numerous preclinical studies over the past decade^2,3,5–9^ raise concerns for the safety, predictability and efficacy of these neural transplants for clinical translation. These low DA yields are in spite of advanced in vitro differentiation protocols that routinely report the absence of PSCs and correct regional fate specification of progenitors (typically> 85% VM OTX2 + FOXA2+, with low off-target cell identities). Such observations indicate extensive expansion of a seemingly low fraction of poorly specified cells within the donor material following implantation, and/or the latent proliferation of quiescent stem cells within the graft. In acknowledgement of their lack of purity, recent studies by us and others have performed transcriptional profiling of hPSC-derived VM progenitor grafts to gain greater insight into the identity of the vast majority of these non-dopaminergic cell types^30,31^. While cells of neural identity form the majority of these grafts, many other cell types are also present^30,31^, for which their impact on graft and/or host function can be detrimental or are currently unknown. Added to this, Tiklova et al.^31^, recently described a high proportion of leptomeningeal-like cells within these grafts, a population that was notably absent from grafts in this present study and likely reflects the impact of subtle differences in in vitro differentiation procedures across research sites that may impact on graft compositions and possible function, and argues the need for further standardisation.

Cell sorting methodologies have been the most widely adopted approach to standardise grafting outcomes—aimed at improving DA proportions and eliminating unwanted cell populations within the donor preparations^7,8,11,12^. Yet outcomes from these studies have been somewhat disappointing, largely due to failure to identify a target protein/gene with restrictive selectivity for DA progenitors, and consequently the inclusion of other cell types that expand after implantation. A further risk of sorting approaches is the risk of undesirable, incorrectly specified and/or highly proliferative cells evading the sorting process, noting recent works reporting up to a 1% error in sorting efficiency^7^. Nevertheless, where advancement in differentiation protocols and cell sorting methodologies have failed to guarantee predictable composition and safety of hPSC-derived neural progenitor grafts, the adoption of suicide-based approaches enables control of hPSC-derived donor cells following their implantation.

Here we highlight the necessity for understanding the growth kinetics of progenitors in order to gain maximal benefit from the FailSafe^TM^ suicide gene-carrying hPSCs for the purpose of transplantation. Similar to previous reports, using the FailSafe^TM^ CDK1-TK hPSC line, we showed that despite implantation of >80% correctly specified VM progenitors, only a small fraction of the graft (<6% of the total cells implanted) were TH + DA neurons at 6 months. Temporal assessment of these transplants revealed that the full complement of the DA neurons was present in the grafts within just 5 weeks after implantation. Surprisingly, beyond this time, grafts continued to exponentially expand over months, with no further increase in DA neurons, and rather a dilution of the density of these functional cells. Not only did this highlight the onset of expansion of the vast majority of unwanted and risk-potential cells, but also warrants consideration for the impact of unnecessarily large neural grafts that displace the underlying host striatum and the possible functionality of the residing medium spiny neurons. Activation of the suicide gene was therefore specifically aimed to coincide with the cell cycle exit of the DA progenitors and their adoption of a post-mitotic TH+ neuronal fate, commencing GCV prodrug treatment at 5 weeks after implantation. Activation of the suicide gene at this time was confirmed to significantly reduce graft size and the relative contribution of unwanted cells without affecting DA neuron yield, their capacity to innervate the host tissue and most importantly reverse motor deficits in Parkinsonian rats. Within these GCV-treated grafts we observed an overall reduction in all other cell populations—inclusive of both neural and non-neural lineages. Most evident however was the significant reduction in the proportion of astroglia and reciprocal increase in neurons, that unsurprisingly reflects neural development whereby gliogenesis succeeds neurogenesis.

While neurons accounted for 39% of cells within the GCV-treated grafts, <25% of these NeuN+ neurons could be phenotyped as DAergic, GABAergic or Cholinergic (noting an absence of adrenergic/noradrenergic and, importantly, serotonergic neurons—a population previously linked to graft-induced dyskinesias in preclinical and clinical studies^32,33^). One is left to wonder whether the remaining NeuN+ neuronal population failed to mature, and therefore are unlikely to have an impact on graft or host function.

Of critical importance was the significant (46%) reduction in non-neuronal cells within the grafts that remained of unknown identity and thereby also unknown functional impact, as well as an >40% decrease in KI67+ proliferative cells. While proliferative cells remained after GCV-treatment, these cells were notably sparse, occurring as singular profiles indicative of their low doubling rate and therefore minimal risk. Furthermore, their close juxtaposition to blood vessels leaves one to question whether these graft-derived progenitors may in fact contribute to the vasculature. Further characterisation, beyond the presented hCOL1A1 immunolabeling, will be required to confirm the identity of these cells. An additional consideration is whether a longer duration of GCV treatment (beyond the present 8-week treatment regime) or intermittent treatment may be required to ablate these remaining proliferative cells, which likely reflect quiescent stem cells appearing after cessation of treatment at 13 weeks, and prior to graft examination at 24 weeks.

A criticism of suicide-based technology for the purpose of improving the safety of transplantation has been the risk of silencing or mutation of the suicide gene and/or diploid loss of heterozygosity within the donor cells^18,34^. To circumvent this, we recently engineered a hPSC line through biallelic targeting which carries a transcriptional link between the suicide gene and a cell cycle essential gene (cyclin-dependent kinase 1). We demonstrated utility of the line to circumvent teratoma formation following hPSC grafts, as well as hPSC-derived retinal pigmental epithelia transplants into the eye^18^. The lack of this FailSafe^TM^ approach likely underpinned the recent failed efforts of Tieng et al.^17^, to eliminate high proportions of proliferative cells following the transplantation of VM progenitors derived from a hPSC line carry a TK gene (driven by the KI67 promoter). It is also plausible that their failed efforts reflected a suboptimal GCV regime. Within the present study, we highlight the importance of understanding the cell cycle kinetics of the proliferative cell population/s targeted for ablation, indicating that while PSC were easily ablated in vitro in the present work and in teratoma focused in vivo studies^14,15,17,18^, the prolonged cell cycle length and proportionately shorter S-phase of neural progenitors (and likely other lineage-restricted progenitors) suggest that longer durations and/or frequency of GCV administration may be required. Added to this is the need to understand the half-life of the pro-drug GCV within the relevant tissue—noting studies reporting undetectable GCV within blood serum at 2 h after intraperitoneal administration^27^, yet present for up to 60 h after intravitreal delivery in the eye^35^. These combined observations of the prolonged VM progenitor cell cycle kinetics, the need for prolonged in vitro GCV treatment to achieve ablation of proliferative neural progenitors, and the short half-life of systemically delivered GCV provided the justification for the adopted 8-week GCV regime. Future studies optimising the duration of GCV treatment, frequency of administration and dose will be required to improve the use of the FailSafe^TM^ suicide system, and require important context-dependent attention.

While the present study demonstrates the capacity of the FailSafe^TM^ suicide hPSC line to improve purity in graft composition and ensure safety, a large proportion of these grafts remained non-DAergic. Fine-tuning to maximize the effect of the FailSafe^TM^ suicide system and further studies involving combined approaches with alternative strategies to selectively ablate unwanted cells and/or protect desired cell populations should be considered for development, to enable maximal control of the suicide-activating drug, targeted at ensuring optimal graft composition, functionality and safety. Added to this remains the need to more rigorously assess the functional capacity for these suicide-gene activated grafts to reverse motor and sensorimotor deficits, through a wider array of relevant behavioural tasks.

With hPSC rapidly advancing to the clinic for PD, careful consideration of the utility and safety of these cells is required. Ensuring safety within the current and proposed trials is of the upmost importance not only for the currently enroled PD patients but for the breadth of application of these cells in other trials and for the treatment of other diseases. Poor regulation and assessment of foetal tissue trials in PD through the 1980s and 1990s created highly variable outcomes and degrees of catastrophe for both the research and clinical community^36^. Local regulatory bodies, such as the FDA, are demanding vast numbers of animals to be grafted with a given cell batch prior to its clinical use, as well as extensive quality control testing focused on cell proliferation, indicating that the concern for tissue overgrowth-associated risks remain^37,38^. Of final consideration will be the need to navigate through the various regulatory barriers required to take a suicide gene-carrying cell line into the clinic. Whilst the precedence has been set in other clinical trials employing T-cell gene therapy to eradicate leukemia and treat graft-versus-host disease^13^, for example, it will nonetheless need readdressing in the context of cell replacement therapy for brain repair.

In conclusion, these findings provide evidence of for a FailSafe^TM^ suicide-based system to improve the composition, purity and safety of hPSC-derived neural grafts for PD without negatively impacting on graft functionality. These findings hold significant implications for future clinical trials involving the transplantation of hPSC-derived neural progenitors.

## Methods

### Cell culture, differentiation and ablation

The H1 human embryonic stem cell line, expressing HSV-TK transcriptionally linked to the CDK1 expression (and subsequently referred to as the FailSafe^TM^ hPSC line), was cultured and differentiated on Laminin-521 (0.5 µg/cm^2^, Biolamina) under xenogeneic-free culture conditions^4^. To initiate differentiation, all cells were exposed to dual-SMAD inhibition using SB431542, (10 µM, R&D Systems, days 0–5, D0–5) and LDN193189 (100–200 nM, 0-11D, Stemgent) to generate neuroectodermal progenitors. To generate VM progenitors suitable for transplantation, cells were subsequently ventralised using Sonic hedgehog C25II (100 ng/ml; R&D systems) and Purmorphamine (2 µM; Stemgent) from D1 to 7, in addition to caudalisation by the canonical WNT agonist CHIR 99021 (3 µM; Stemgent, D3–13D). At D11, cells were transitioned into a Maturation media consisting of NBB27 supplemented with GDNF (20 ng/ml; R&D systems), BDNF (20 ng/ml; R&D Systems), TGFβ3 (1 ng/ml; PeproTech), DAPT (10 µM; Sigma-Aldrich), ascorbic acid (200 nM; Sigma-Aldrich), and dibutyryl cAMP (0.05 mM; Tocris Bioscience)^4^.

For in vitro ablation of proliferative PSCs or differentiating VM neural progenitors, cells were exposed to GCV (GCV, 10 µM, Roche) for either 5 or 10 days, with culture medium (±GCV) changed daily. For transplantation, D19 (or D25) VM progenitors were dissociated in Accutase (Stem Cell Technologies) and resuspended into maturation media supplemented with ROCK inhibitor (Y27632, 10 µM, Sigma-Aldrich) at 125,000 cells/µl.

### Cumulative EdU labelling for cell cycle analysis

For assessment of cell cycle kinetics, total cell cycle (Tc) and S-phase length (Ts) were determined using the Click-iT EdU kit (Life Technologies). Briefly, cultures (PSCs & VM progenitors) were seeded at 5–7 × 10^4^ cells/cm^2^ in 96-well plates pre-coated with Laminin-521 (0.5 µg/cm^2^). The following day, cultures were changed with appropriate media and 5-ethynyl-2′-deoxyuridine (EdU; 5 μM) was added to wells sequentially at 12, 9, 6, 4, 3, 2, 1 and 0.5 h (hr) time points for PSCs and 20, 16, 8, 6, 4, 3, 2, 1 and 0.5 hrs for NPCs prior to simultaneous fixation in 4% (w/v) PFA. Cultures were permeabilised with 0.3% (v/v) Triton-X for 20 min before undergoing Click-iT reaction as per manufacturer’s instructions (Click-iT EdU kit). Azide-bound Alexa-488 or −594 was used to visualise incorporation of Edu followed by counterstaining with Hoechst 33342 (5 µM, 10 min). Adopting previously described methodology^22,23,39^, to calculate cell cycle kinetics the % of Edu+ cells were quantified at each fixation time point for both cell types. The time taken for the growth phase to plateau (0–8 h or 0–18 h for PSC and NPCs, respectively) represented the peak of EdU reactivity (the point at which all EdU+ cells have completed a full cell cycle and re-entered S phase). This peak demarcated the Tc-Ts value, where Tc denotes total cell cycle length and Ts the total length of S phase. Ts was then calculated by extrapolating the linear regression model fit during the growth phase period to the *x*-axis intercept (Supplementary Fig. 2).

### Surgical procedures

All animal procedures were performed in agreement with the Australian National Health and Medical Research Council’s published Code of Practice for the Use of Animals in Research, and approval granted by The Florey Institute of Neuroscience and Mental Health Animal Ethics committee. Animals (of either sex) were group housed on a 12:12-h light/dark cycle with ad libitum access to food and water. For studies involving behavioural assessment, groups included 8–10 rats, while assessments of histology only (e.g. for assessment of grafts at earlier time points) employed smaller group sizes of 4–6 rats.

Surgeries were performed on athymic (CBH^rnu^) nude rats under 2–5% isoflurane anaesthesia. Rats received unilateral injections (3.5 µl) of 6-OHDA (3.5 μg/μl free base dissolved in a solution of 0.2 mg/ml L-ascorbic acid in 0.9% w/v NaCl) into the medial forebrain bundle (MFB, 3.4 mm posterior, and 1.3 mm lateral to bregma, and 6.8 mm below the dura surface) using a 10 ml Hamilton syringe fitted with a glass capillary. At 8 weeks after 6-OHDA lesioning animals received unilateral cell grafts (1 µL, 125,000 cells/µl), at the following stereotaxic coordinates: 0.5 mm anterior, 2.5 mm lateral to bregma and 4.0 mm below the dura surface. Daily intraperitoneal (i.p.) injections of GCV (50 mg/kg) were administered to a subset of animals from 5–13 weeks or 14–22 weeks after transplantation.

In a separate cohort of grafted animals, GCV was administered from 5 to 10 weeks and animals (including non-GCV-treated graft controls) were killed at 10 weeks to enable assessment of local inflammation during periods of ongoing cell death.

### Behavioural analysis

Testing for rotational asymmetry, induced by D-amphetamine sulfate (3.5 mg/kg i.p., Tocris Biosciences), was conducted 4 weeks after 6-OHDA administration to confirm motor deficits in unilateral lesioned rats. All animals displaying a functional deficit (>300 rotations in 60 min) were ranked in order of the percentage rotational asymmetry and evenly distributed across the four treatment groups: (i) Lesion only (subsequently referred to as Lesion), (ii) Lesion + GCV (GCV), (iii) VM progenitor cell graft (Cells), (iv) Cells + GCV administered from 5 to 13 weeks (Cells + GCV), (v) Cells + GCV administered from 14 to 22 weeks (referred to as Cells+ late GCV). To assess functional integration of transplanted cells, behavioural testing was repeated at 24 weeks after grafting.

### Tissue processing and histochemistry

For confirmation of appropriate in vitro specification of VM neural progenitors, cultures were fixed using paraformaldehyde (PFA, 4%, 10 min) at varying time points during the differentiation (0, 8, 11, 16, 19, 25D). For assessment of graft survival, composition and integration, animals were killed by an overdose of sodium pentobarbitone (100 mg/kg) at 5, 10, 20 or 24 weeks after transplantation and transcardially perfused with 4% PFA. Brains were cryosectioned (40 µM; 12 series) on a freezing microtome. Immunohistochemistry was performed on free-floating brain sections. The tissue was incubated overnight with primary antibodies diluted in PBS containing 5% normal serum and 0.25% Triton X-100 (Amresco, USA). Primary antibodies and dilutions are shown in Supplementary Table 1. Detection of the primary–secondary antibody complexes was through peroxidase driven precipitation of di-amino-benzidine (DAB), or conjugation of a fluorophore. Secondary antibodies generated in donkey were applied for 2 h at room temperature at a dilution of 1:400 for fluorescent detection using 488, 549 or 649 conjugated anti-mouse, anti-chicken, anti-rabbit, antisheep or anti-goat (Jackson ImmunoResearch). Chromogenic detection of antibody-DAB complex was carried out using biotin-conjugated donkey anti-rabbit (1:500, 2 h; Jackson ImmunoResearch) followed by peroxidase conjugated streptavidin (1 h, Vectastain ABC kit, Vector laboratories) and incubation with DAB (0.5 mg/ml, 5 min), which was precipitated by addition of 1% w/v H2O2. Fluorescently labelled sections were cover-slipped with fluorescent mounting media (Dako) and chromogenic labelled sections dehydrated in alcohol and xylene and cover-slipped with DePeX mounting media (BDH Chemicals, UK).

### Microscopy and quantification

Brightfield images of chromogenic staining were captured using a Leica Microscope DM6000 microscope. Fluorescent images were obtained on a Carl Zeiss Axio Observer Z.1 epifluorescence or Carl Zeiss LSM780 confocal microscope. Images captured at 20× were used to quantify the total number of DAPI+, OTX2+, FOXA2+, HNA+, KI67+, EdU+, NEUN+, CC1+, SOX9+, GABA+, ChAT+, Iba1+, GFAP+ and RECA+ cells, either in culture or within the graft. The total cell counts were made across the rostro-caudal axis of the graft (from 1:12 series), spanning ~4–6 sections per graft. Positive hCOL1A1 staining was confirmed by expression in hPSC-derived teratoma (Supplementary Fig. 8B). PSA-NCAM expression was used to delineate the graft boundaries and estimate graft volume according to Cavalieri’s principle^40^. Quantification of tyrosine hydroxylase (TH+) DA neurons was performed on live images. Assessment of GIRK2+ and Calbindin+ cells within the grafts were quantified based on co-localisation with TH+ from acquired confocal images.

Graft-derived TH+ fibre density was assessed only in animals that showed a robust behavioural deficit (>5 rotation/min) at 2 weeks after 6-OHDA lesioning and were confirmed to have ≥80% TH+ cell loss within the midbrain at 24 weeks (as depicted in Supplementary Fig. 4A). These brains were selected to minimise the chance of including residual host TH+ fibres within the quantifications. Fibre density was assessed within the dorsolateral, ventrolateral and medial striatum (as illustrated in Supplementary Fig. 4B), across the rostro-caudal axis of the nuclei, spanning six sections (2.4 mm). For each of the three sites within the striatum, and at each of the six rostro-caudal levels, 10 z-stack sections (1 µm per section) were obtained and compressed. TH+ fibres were isolated on colour inverted images using the colour range tool on Photoshop (Adobe). Data are expressed as percentage of immunoreactive pixels. All areas were captured in triplicate with conserved settings.

### Statistical analysis

All behavioural testing and histological quantification were conducted by researchers blinded to experimental groups. All data are presented as mean ± SEM. Statistical tests employed (one-way ANOVA, two-way ANOVA, Student’s *t* tests), and number of animals/groups for each assessment are stated in the figure legends. All statistical analyses were performed using GraphPad Prism and alpha levels of *p* < 0.05 were considered significant (*p < 0.05, **p < 0.01, ***p < 0.001).

### Reporting summary

Further information on research design is available in the Nature Research Reporting Summary linked to this article.

## Supplementary information

Supplementary Information

Reporting Summary

## Data Availability

The authors declare that the data supporting the findings of this study are presented within the paper and its supplementary information files. Source data are provided with this paper.

## Source data

Source Data
