# Supplementary material for: Human stem cells harboring a suicide gene improve the safety and standardisation of neural transplants in Parkinsonian rats
Source: Nat Commun. 2021 May 27;12:3275. doi: 10.1038/s41467-021-23125-9 (PMC8160354; doi:10.1038/s41467-021-23125-9)
Supplement: Supplementary file 1 — Supplementary Information [file 41467_2021_23125_MOESM1_ESM.pdf]

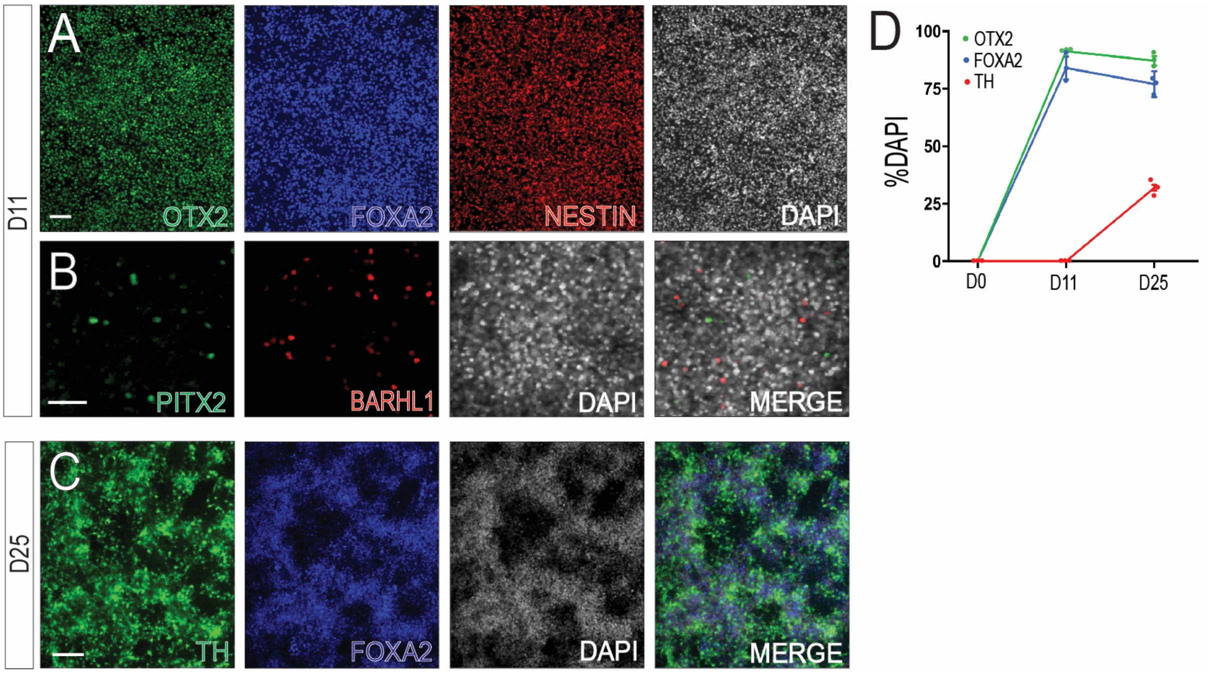

Supplementary Figure 1: Validation of VM specification of the FailSafe™ suicide gene-carrying hPSC into midbrain progenitors and DA neurons

(A-B) Efficient differentiation of the FailSafe™ CDK1-TK hPSC into VM progenitors was confirmed by high co-expression of OTX2 (green), FOXA2 (blue) and NESTIN (red) by D11 (A), and a low proportion of neighbouring, off-target subthalamic nucleus-like progenitors - identified by PITX2+ (green) and BARHL1+ (red) cells, B). (C) Representative photomicrographs illustrating the capacity of the correctly specified VM progenitors to mature into TH+ (green) FOXA2+ (blue) DA neurons by D25. (D) Quantification of expression of cardinal VM markers OTX2, FOXA2 and TH confirmed efficient VM differentiation at D11 and D25. Data are presented as mean values +/- SEM, One-way ANOVA with Tukey's correction for multiple comparisons; n=3 independent cultures/time point. Scale bars = 100µm. Abbreviations: D, days in differentiation.

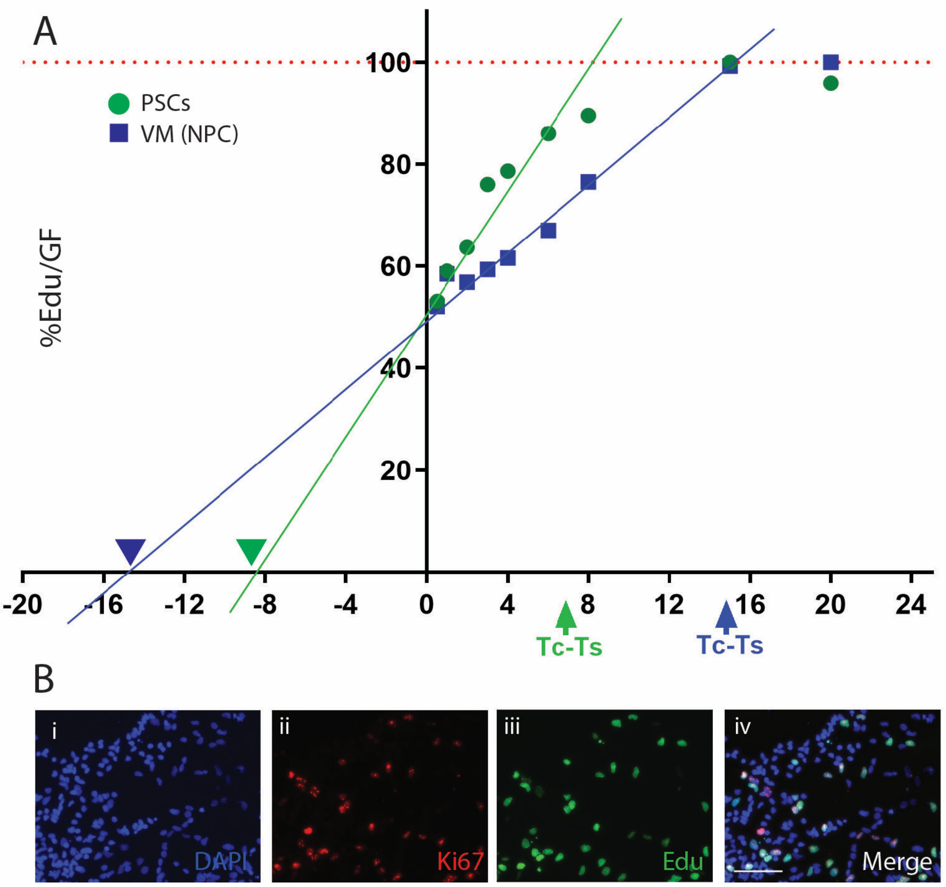

Supplementary Figure 2: Comparing the cell cycle kinetics of human PSC and VM progenitor in vitro.

(A) Plots of EdU+ cycling progenitors normalised to the growth fraction (GF). GF is defined as the maximal percentage of EdU+/Ki67+ of total Ki67+ proliferating cells. Arrows represent the average time taken to reach EdU saturation (red dotted line) and demarcates Tc-Ts where Tc is the total cell cycle length and Ts is the time in S phase. The x-intercept is Ts (arrow heads) for VM progenitors (blue) and PSCs (green). Data is from 3 independent experiments in duplicate. (B) Representative photomicrograph of (i) DAPI (blue), (ii) Ki67 (red), (iii) and EdU (green) within a representative VM differentiating culture at D10. n=3 independent cultures. Scale bar = 100 µm. Abbreviations: EdU, 5-ethynyl-2'-deoxyuridine; Tc, total cell cycle length; Ts, total S phase length; VM, ventral midbrain.

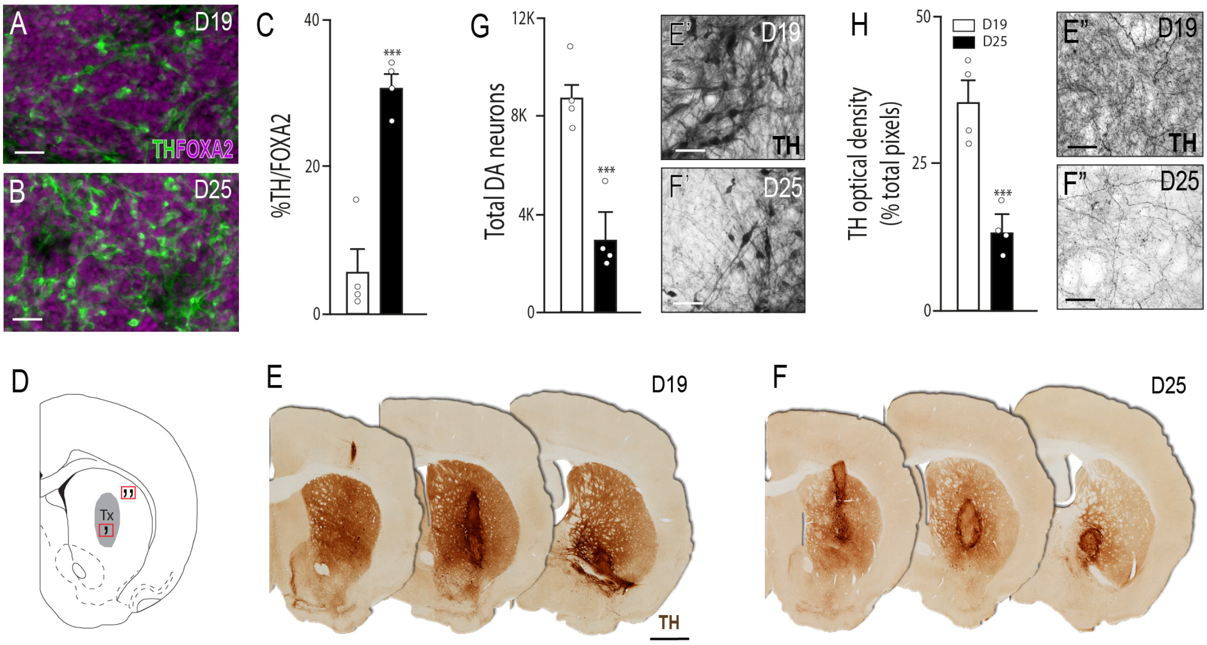

Supplementary Figure 3: Younger hPSC-derived progenitors generate superior grafts enriched with TH+ DA neurons and enhanced capacity to innervate the host striatum.

(A) Representative images of hPSC VM progenitors at D19 and (B) D25 of differentiation, illustrating the increase in DA neurons co-expressing TH+ (green) and FOXA2 (magenta) with time. (C) Quantification of TH+ neurons, as a proportion of FOXA2+ VM cells *in vitro*. (D) Schematic, unilateral coronal section of the rodent brain, at the level of the striatum, illustrating sampling sites where images of the cells within the grafts were captured (‘) and density of TH+ innervation (“). (E) Representative overview of a graft at 26 weeks after implantation of D19 or (F) D25 hPSC-derived VM progenitors. (E’,F’) Representative images illustrating the increase number, and density, of TH+ DA neurons within grafts derived from D19 versus D25 progenitors, as well as (E”,F”) elevated TH+ striatal innervation. (G) Quantification of TH+ DA neurons with grafts derived from D19 or D25 VM progenitors and (H) density of TH+ fibers within the dorsolateral striatum. (C) n=4 independent cultures, (G,H) n=4 grafts/group. Data are presented as mean values +/- SEM. (C,G,H) Student’s t test, *** p<0.001.

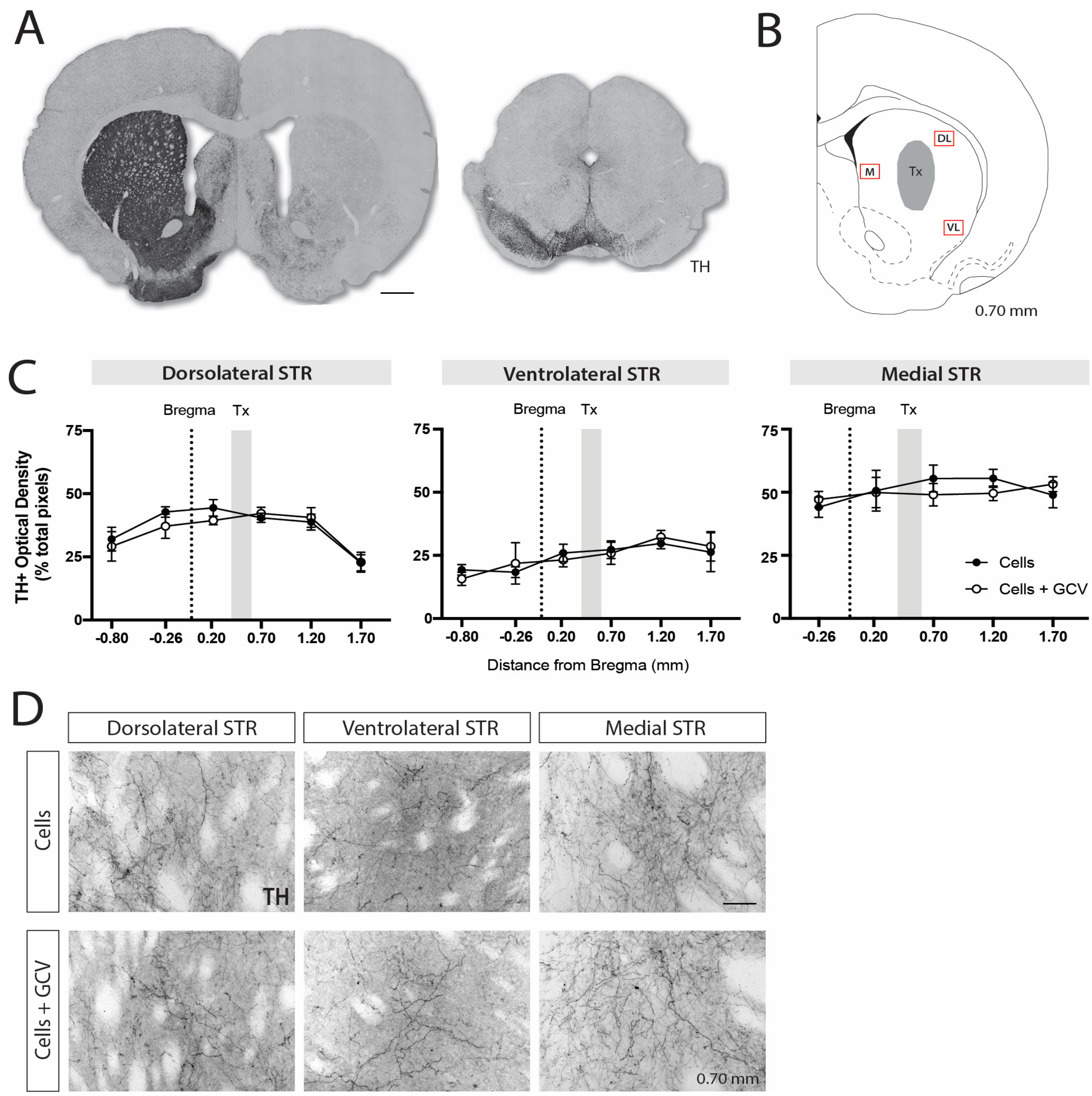

Supplementary Figure 4: GCV-treated grafts maintain their capacity to innervate the host striatum.

(A) Representative images demonstrating successful unilateral 6-OHDA-induced ablation of the host midbrain dopaminergic circuitry, as reflected by the loss of TH+ fibers within the striatum and cells within the ventral midbrain. (B) Schematic illustration highlighting the sampling sites around the graft at which TH+ fiber density assessments were analysed, depicted here at a single level of the striatum. (C) Quantitative assessment of TH-immunoreactive pixels (as a % of total pixels) within the dorsolateral, ventrolateral and medial striatum (across the rostro-caudal axis of the nucleus). (D) Representative photomicrographs of TH labeling within the striatal sampling sites from a representative Cells and Cells + GCV grafted animal at 26 weeks. Data are presented as mean values +/- SEM; n=4 grafts/group. Scale bars: (A) 1mm, (D) 100µm. Abbreviations: 6-OHDA; 6-hydroxydopamine; DL, dorsolateral striatum; GCV, ganciclovir; M, medial striatum; STR, striatum; TH, tyrosine hydroxylase; Tx, transplant; VL, ventrolateral striatum.

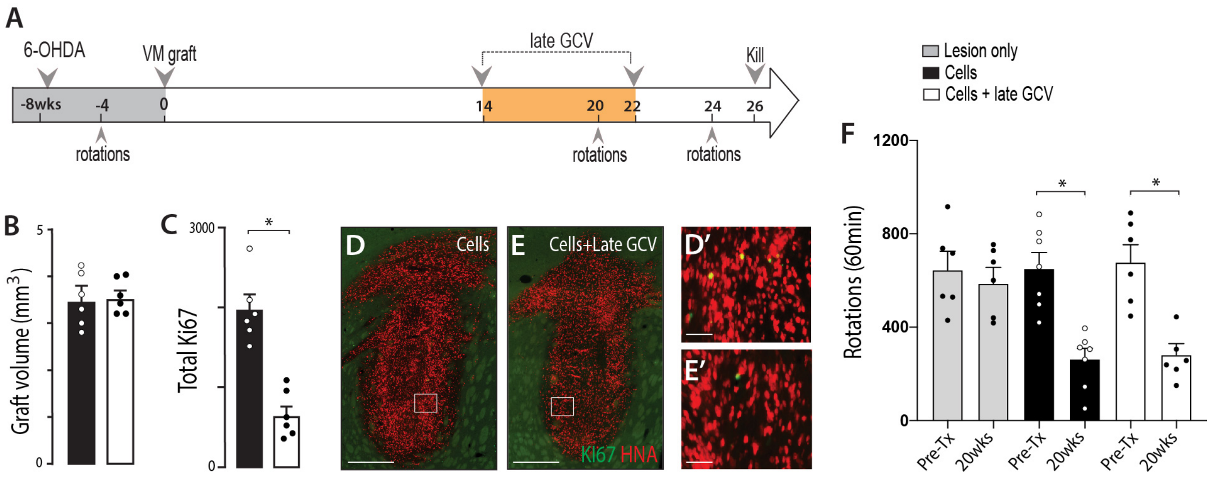

Supplementary Figure 5: Delayed GCV treatment reduces residual proliferative cells and demonstrates the ability of grafts to retain their functional capacity.

(A) Experimental design for late GCV administration in grafted animals. (B) Late GCV treatment (from 14-22weeks) had no impact on graft size, (C) yet significantly reduced the number of residual KI67+ proliferative cells within the graft. (D-E) Representative images of the grafts showing reduced KI67+ cells following late GCV treatment (E), compared to non-GCV treatment (D). (D'-E') Higher magnificantion images taken from D and E. (F) Behavioural testing at 20 weeks after transplantation, and during ongoing GCV-treatment, revealed that neither the drug nor presence of suicide-induced dying cells within the grafts had any detrimental impact on the functionality of the DA neurons, with comparable improvements in rotational asymmetric testing for Cells vs Cells + Late GCV grafted animals. Data are presented as mean values +/- SEM; Multiple Student t-tests, * $p<0.05$. Scale n=6 grafts/group. bars: (D,E) 500µm; (D',E') 100µm. Abbreviations: 6-OHDA; 6-hydroxydopamine; GCV, ganciclovir; VM, ventral midbrain, HNA, human nuclear antigen; Pre-Tx, pre-transplantation; wks, weeks.

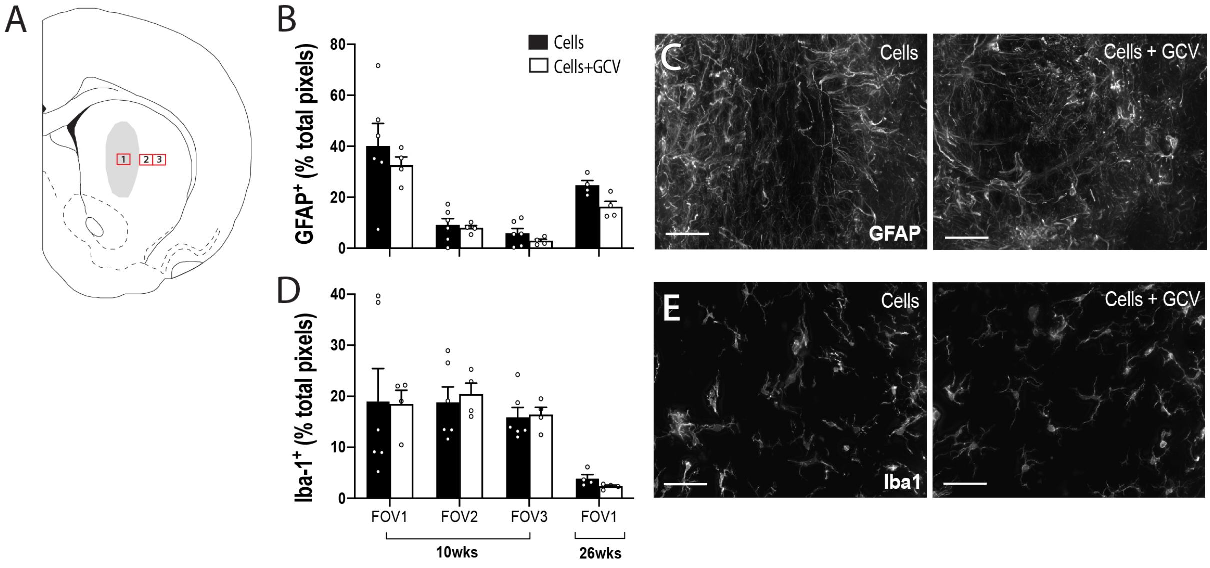

Supplementary Figure 6: Suicide-induced cell ablation does not exacerbate local inflammation.

(A) Schematic illustration highlighting the sampling sites (0.7mm anterior to Bregma) for estimates of local inflammation within the graft (Box 1), at the graft-host interface (Box 2) and more distal to the graft (Box 3). (B) Quantitative assessment of the density of GFAP-immunoreactive pixels (as a proportion of total pixels) at the 3 pre-determined fields of view (FOV, depicted in panel A) at 10 weeks after grafting, noting the increase in reactive astrocyte density overlying the graft core (FOV1) and reducing at more distal sites, yet remaining unchanged between GCV- and non-GCV treated grafts. (C) Representative photomicrographs showing GFAP labeling at FOV1 at 10 weeks post-grafting. (D) Quantitative assessment of the proportion of Iba1-immunoreactive pixels (as a proportion of total pixels) within and surrounding the graft site. Note, levels of inflammatory cells notably reduced by 26 weeks (at FOV1). (E) Sample images of Iba1+ staining at FOV1 (at 10 weeks) within Cells and Cells + GCV treated grafts. Data are presented as mean values +/- SEM; Cells: n=6; Cells+GCV: n=4. Scale bar: 100µm. Abbreviations: FOV, field of view; GCV, ganciclovir.

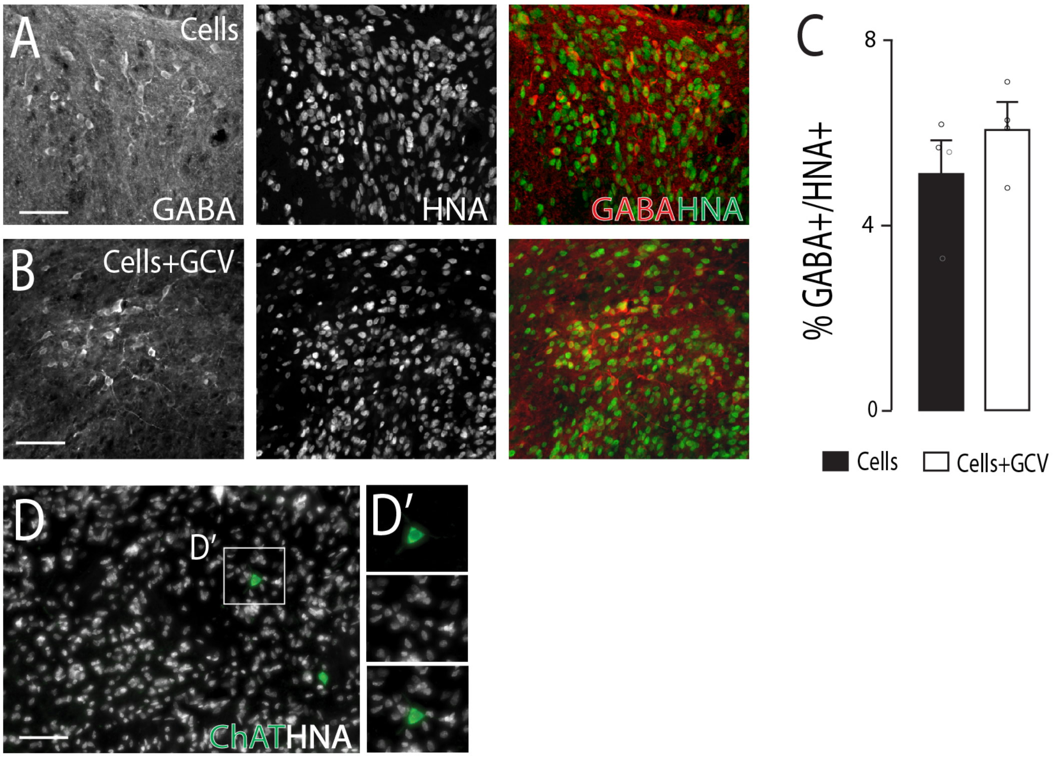

Supplementary Figure 7: Neuronal phenotyping of FailSafe^{TM} hPSC-derived VM progenitor grafts.

(A-B) Representative images showing HNA+ graft-derived GABAergic neurons within grafts of FailSafe^{TM} hPSC-derived VM progenitors in the absence (A) and presence (B) of GCV. GABA (red), HNA (green). (C) Quantification of the proportion of GABA+ neurons within the graft (GABA+HNA+/HNA+). (D) Representative image of a rare ChAT+ (green) cholinergic neurons within a graft. Data are presented as mean values +/- SEM; n=4 grafts/group. Scale bars: (A,B,D) 100µm. Abbreviations: ChAT, Choline acetyltransferase; GABA, Gamma aminobutyric acid; GCV, ganciclovir; HNA, human nuclear antigen.

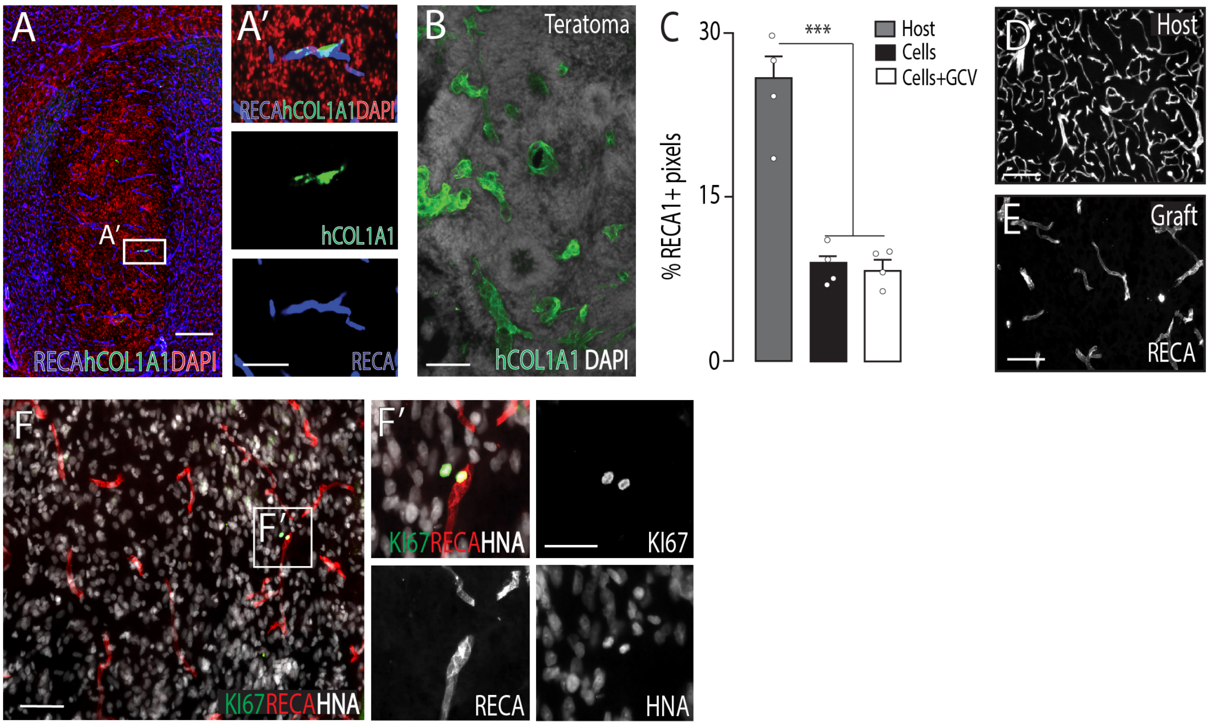

Supplementary Figure 8: Assessment of non-neural cells within FailSafe™ hPSC-derived VM progenitor grafts.

(A) Representative FailSafe™ hPSC-derived VM progenitor graft at 26 weeks immunolabeled for DAPI (red), RECA (blue), and human-specific COL1A1 (green), illustrating that the majority of vascularization of the graft was of host, and not graft, origin. (B) Representative image illustrating hCOL1A1 (green) labeling within blood vessel-like structures within a FailSafe™ hPSC-derived teratoma (in the absence of GCV). (C) Quantification of RECA-immunoreactive pixels within the grafts showing that, while blood vessels were present, they were significantly less dense than in the host tissue, yet not different between the graft groups (± GCV treatment). (D-E) Photomicrographs showing the density of host-derived RECA+ blood vessels within the host (D) and graft tissue (E). (F) Representative image showing the rare presence of a KI67+ (green) proliferative cell within a FailSafe™ hPSC-derived VM progenitor graft (treated with GCV) at 26 weeks. These rare KI67+ cells were most often observed within close proximity to the hosts (RECA, red) vascular supply. (F') Higher magnification of image depicted in F. Data are presented as mean values +/- SEM; One-way ANOVA with Tukey's correction for multiple comparisons; n=4 animals/group. ***$p < 0.001$. Scale bars: (A) 500µm, (A',B) 200µm, (D,E,F) 100µm, (F') 50µm. Abbreviations: GCV, ganciclovir; HNA, human nuclear antigen; hCOL1A1, human collagen type I alpha 1; RECA, rat endothelial cell antigen-1.

Supplementary Table. 1. Primary antibodies – species, source, identifier and dilution

Antibody	Species	Source	Identifier	Dilution
5HT	Rabbit	Immunostar	20080	1:1000
BARHL1	Rabbit	Novus Biologicus	NBP1-86513	1:200
Calbindin	Mouse	Swant	CB300	1:1000
Adenomatous polyposis coli, clone CC1	Mouse	Abcam	ab16794	1:200
ChAT	Goat	Millipore	AB144P	1:100
COL1A1	Sheep	R&D Systems	AF6220	1:100
DAPI	-	Sigma Aldrich	D8417	1:5000
DBH	Mouse	Millipore	MAB308	1:5000
FOXA2	Goat	Santa Cruz	sc-6554	1:200
GABA	Rabbit	Sigma	A2052	1:1000
GFAP	Rabbit	DAKO	ZO334	1:1000
GIRK2	Rabbit	Alomone Labs	APC-OO6	1:500
HNA	Mouse	Millipore	MAB1281	1:300
Iba1	Rabbit	WAKO	019-19741	1:1000
KI67	Rabbit	ThermoFisher	LBVRM-9106-S1	1:1000
NEUN	Rabbit	R&D Systems	ab104225	1:1500
NESTIN	Mouse	Millipore	MAB1259	1:1000
OTX2	Goat	R&D Systems	RDSAF1979	1:500
PH3	Rat	Abcam	AB10543	1:1000
PITX2	Sheep	R&D Systems	AF7388	1:200
hPSA-NCAM	Mouse	Santa Cruz	sc-106	1:500
RECA	Mouse	Abd Serotec	MCA970R	1:5000
SOX9	Rabbit	Abcam	Ab185966	1:500
TH	Rabbit	Pel-freeze	P40101-0	1:1000
TH	Sheep	Pel-freeze	P60101-0	1:800
TUJ (βIII-tubulin)	Mouse	Promega	G712A	1:1000
