# Supplementary material for: Human stem cells harboring a suicide gene improve the safety and standardisation of neural transplants in Parkinsonian rats
Source: Nat Commun. 2021 May 27;12:3275. doi: 10.1038/s41467-021-23125-9 (PMC8160354; doi:10.1038/s41467-021-23125-9)
Supplement: Supplementary file 2 — Reporting Summary [file 41467_2021_23125_MOESM2_ESM.pdf]

nature research

Corresponding author(s): Clare Parish

Last updated by author(s): Mar 3, 2021

Reporting Summary

Nature Research wishes to improve the reproducibility of the work that we publish. This form provides structure for consistency and transparency in reporting. For further information on Nature Research policies, see our Editorial Policies and the Editorial Policy Checklist.

Statistics

For all statistical analyses, confirm that the following items are present in the figure legend, table legend, main text, or Methods section.

n/a	Confirmed	
☐	☒	The exact sample size (n) for each experimental group/condition, given as a discrete number and unit of measurement
☐	☒	A statement on whether measurements were taken from distinct samples or whether the same sample was measured repeatedly
☐	☒	The statistical test(s) used AND whether they are one- or two-sided<br>*Only common tests should be described solely by name; describe more complex techniques in the Methods section.*
☒	☐	A description of all covariates tested
☐	☒	A description of any assumptions or corrections, such as tests of normality and adjustment for multiple comparisons
☐	☒	A full description of the statistical parameters including central tendency (e.g. means) or other basic estimates (e.g. regression coefficient) AND variation (e.g. standard deviation) or associated estimates of uncertainty (e.g. confidence intervals)
☐	☒	For null hypothesis testing, the test statistic (e.g. F, t, r) with confidence intervals, effect sizes, degrees of freedom and P value noted<br>*Give P values as exact values whenever suitable.*
☒	☐	For Bayesian analysis, information on the choice of priors and Markov chain Monte Carlo settings
☒	☐	For hierarchical and complex designs, identification of the appropriate level for tests and full reporting of outcomes
☒	☐	Estimates of effect sizes (e.g. Cohen's d, Pearson's r), indicating how they were calculated

Our web collection on statistics for biologists contains articles on many of the points above.

Software and code

Policy information about availability of computer code

Data collection	Adobe Photoshop (21.2.5 release)
Data analysis	Statistical significance was estimated with t-tests or one way ANOVA, using p-values (GraphPad prism software v8)

For manuscripts utilizing custom algorithms or software that are central to the research but not yet described in published literature, software must be made available to editors and reviewers. We strongly encourage code deposition in a community repository (e.g. GitHub). See the Nature Research guidelines for submitting code & software for further information.

Data

Policy information about availability of data

All manuscripts must include a data availability statement. This statement should provide the following information, where applicable:

- Accession codes, unique identifiers, or web links for publicly available datasets
- A list of figures that have associated raw data
- A description of any restrictions on data availability

The authors declare that the data supporting the findings of this study are presented within the paper [and its supplementary information files]. Raw data sets are available upon request.

Field-specific reporting

Life sciences study design

All studies must disclose on these points even when the disclosure is negative.

Sample size	No statistical methods were used to predetermine sample sizes but our sample sizes are similar to those reported in our previous publications (Gantner, de Luzy et al., 2020; de Luzy et al., 2019; Niclis et al., 2017- cited within the reference list)
Data exclusions	no data were excluded from analysis
Replication	Reproducibility was ensured by sampling from multiple biological replicates. In vitro experiments were performed on >3 independent experiments for all conditions and for in vivo studies, multiple (n>7 rats or mice were included per group). No results are included that were not observed in multiple experiments.
Randomization	For in vivo studies, animals were tested for amphetamine-induced rotational asymmetry. Animals showing greater than 300 rotations/hour were ranked and evenly assigned across the 4 treatment groups such that baseline behaviour (prior to transplantation) was not different between groups. Details in methods (line 392) Rats were tested for amphetamine-induced rotational asymmetry. Animals showing greater than 300 rotations/hour were ranked and evenly assigned across the 4 treatment groups such that baseline behaviour (prior to transplantation) was not different between groups.<br>For in vitro studies, seeding of pluripotent stem cells or differentiating VM progenitors was consistent across all wells (with cells originating from a common cell suspension) and therefore all wells considered equal in composition prior to any treatment. Technical replicates (n=3 wells/treatment) were performed to allow for any subtle 'between well' variability. Noting all in vitro experiments were also repeated on >3 independent experiments.
Blinding	For behavioural testing and quantification of histological readouts, researchers were blinded to the experimental conditions.

Reporting for specific materials, systems and methods

We require information from authors about some types of materials, experimental systems and methods used in many studies. Here, indicate whether each material, system or method listed is relevant to your study. If you are not sure if a list item applies to your research, read the appropriate section before selecting a response.

Materials & experimental systems

n/a	Involved in the study	
☐	☒	Antibodies
☐	☒	Eukaryotic cell lines
☒	☐	Palaeontology and archaeology
☐	☒	Animals and other organisms
☒	☐	Human research participants
☒	☐	Clinical data
☒	☐	Dual use research of concern

Methods

n/a	Involved in the study	
☒	☐	ChIP-seq
☒	☐	Flow cytometry
☒	☐	MRI-based neuroimaging

Antibodies

Antibodies used

#5HT Rabbit Immunostar #20080 1:1000
BARHL1 Rabbit Novus Biologicus #NBP1-86513 1:200
Calbindin Mouse Swant #CB300 1:1000
Adenomatous polyposis coli, clone CC1 Mouse Abcam #ab16794 1:200
BARHL1 Rabbit Novus Biologicals #NBP1-B6513 1:200
ChAT Goat Millipore #AB144P 1:100
COL1A1 Sheep R&D Systems #AF6220 1:100
DAPI - Sigma Aldrich #D8417 1:5000
DBH Mouse Millipore #MAB308 1:5000
FOXA2 Goat Santa Cruz #sc-6554 1:200
GABA Rabbit Sigma #A2052 1:1000
GFAP Rabbit DAKO #ZO334 1:1000
GIRK2 Rabbit Alomone Labs #APC-OO6 1:500
HNA Mouse Millipore #MAB1281 1:300
Iba1 Rabbit #WAKO 019-19741 1:1000
KI67 Rabbit ThermoFisher #LBVRM-9106-S1 1:1000
NEUN Rabbit R&D Systems #ab104225 1:1500
NESTIN Mouse Millipore #MAB1259 1:1000
OTX2 Goat R&D Systems #RDSAF1979 1:500
PH3 Rat Abcam #AB10543 1:1000
PITX2 Sheep R&D Systems #AF7388 1:200

	hPSA-NCAM Mouse Santa Cruz #sc-106 1:500<br>RECA Mouse Abd Serotec #MCA970R 1:5000<br>SOX9 Rabbit Abcam #Ab185966 1:500<br>TH Rabbit Pel-freeze #P40101-0 1:1000<br>TH Sheep Pelfreeze #P60101-0 1:800<br>TUJ (III-tubulin) Mouse Promega #G712A 1:1000
Validation	The specificity of primary antibodies were validated by manufacturers (PelFreez Biologicals, Millipore, Santa Cruz, ThermoFisher, R&DSystems, Promega and Abcam) and by determining the specificity of signal using regions of the tissue that are negative for the expression of the protein of interest and/or human specificity, and from our previous published experience (Gantner, de Luzy et al., 2020; de Luzy et al., 2019; Somaa et al., 2017).

Eukaryotic cell lines

Policy information about cell lines

Cell line source(s)	The H1 human embryonic stem cell line, sourced from WiCell (USA)), was used for the derivation of the human ESC CDK1-Thymidine kinase homozygous cell line, designated as H1_CDK1-TKhom (according to the original publication of the cell line - See reference Liang et al., 2018).
Authentication	Cell line was authenticated by the provider (co-author A. Nagy) and described in the original publication of the cell line (see reference Liang et al., 2018). At the time of the present study the cell line was confirmed to be karyotype normal.
Mycoplasma contamination	The cell line routinuely tested negative for mycoplasma contamination using Mycoplasma qPCR Detection Kit (Sigma)
Commonly misidentified lines (See ICLAC register)	No commonly misidentified cell lines were used

Animals and other organisms

Policy information about studies involving animals; ARRIVE guidelines recommended for reporting animal research

Laboratory animals	Adult male and female, athymic ''nude'' rats (8-12 weeks of age at the commencement of the study) were purchased from Animal Resource Services, Perth Australia (CBH-rnu)
Wild animals	This study did not involve wild animals
Field-collected samples	This study did not involve field-collected samples
Ethics oversight	All animal procedures were performed in agreement with the Australian National Health and Medical Research Council's published Code of Practice for the Use of Animals in Research, and approval granted by The Florey Institute of Neuroscience and Mental Health Animal Ethics committee.

Note that full information on the approval of the study protocol must also be provided in the manuscript.
